# CaCu_3_Ti_4_O_12_ Perovskite Materials for Advanced Oxidation Processes for Water Treatment

**DOI:** 10.3390/nano13142119

**Published:** 2023-07-20

**Authors:** Elissa Makhoul, Madona Boulos, Marc Cretin, Geoffroy Lesage, Philippe Miele, David Cornu, Mikhael Bechelany

**Affiliations:** 1 Institut Européen des Membranes, IEM, UMR 5635, Centre National de la Recherche Scientifique (CNRS), University Montpellier, ENSCM, Place Eugène Bataillon, 34095 Montpellier, France; elissa.makhoul@hotmail.com (E.M.); marc.cretin@umontpellier.fr (M.C.); geoffroy.lesage@umontpellier.fr (G.L.); philippe.miele@umontpellier.fr (P.M.); david.cornu@umontpellier.fr (D.C.); 2 Laboratoire de Chimie Physique des Matériaux (LCPM/PR2N), EDST, Faculté des Sciences II, Département de Chimie, Université Libanaise, Fanar P.O. Box 90656, Lebanon; madonaboulos@hotmail.com; 3 Institut Universitaire de France, 1 rue Descartes, CEDEX 05, 75231 Paris, France; 4 Gulf University for Science and Technology (GUST), West Mishref, Hawalli 32093, Kuwait

**Keywords:** water pollution, water treatment, electro-oxidation, sulfate activation, perovskite, CaCu_3_Ti_4_O_12_

## Abstract

The many pollutants detected in water represent a global environmental issue. Emerging and persistent organic pollutants are particularly difficult to remove using traditional treatment methods. Electro-oxidation and sulfate-radical-based advanced oxidation processes are innovative removal methods for these contaminants. These approaches rely on the generation of hydroxyl and sulfate radicals during electro-oxidation and sulfate activation, respectively. In addition, hybrid activation, in which these methods are combined, is interesting because of the synergistic effect of hydroxyl and sulfate radicals. Hybrid activation effectiveness in pollutant removal can be influenced by various factors, particularly the materials used for the anode. This review focuses on various organic pollutants. However, it focuses more on pharmaceutical pollutants, particularly paracetamol, as this is the most frequently detected emerging pollutant. It then discusses electro-oxidation, photocatalysis and sulfate radicals, highlighting their unique advantages and their performance for water treatment. It focuses on perovskite oxides as an anode material, with a particular interest in calcium copper titanate (CCTO), due to its unique properties. The review describes different CCTO synthesis techniques, modifications, and applications for water remediation.

## 1. Introduction

Environmental pollution refers to the contamination or degradation of the natural environment, including air, water, soil and ecosystems, by human activities. It results from the release into the environment of harmful substances that can cause harm to human health, wildlife and the natural environment. Environmental pollution, particularly water pollution, is a significant issue worldwide and its effects can be severe and long-lasting [1,2,3,4]. Pharmaceutical products contribute to the water pollution problem. These pollutants, categorized as emerging contaminants, raise particular alarm due to their long persistence and potential effects on the environment and human health, even at low concentrations [5,6,7,8]. Therefore, the topic of this review is advanced oxidation processes (AOPs) able to degrade any organic micro pollutants such as drugs, pesticides, personal care products, dyes, per-and polyfluoroalkyl substances (PFAS), etc., without generating any harmful by-product [9,10]. Moreover, AOP processes can be combined to obtain a synergetic effect. In particular, electro-oxidation or photocatalysis can be coupled with sulfate-radical-based AOP to increase the generation of reactive species and consequently to improve the efficiency and selectivity for target contaminants [11,12]. The choice of anode material is crucial for sulfate activation and charge transfer in the electrocatalysis system. Perovskite can serve as an anode material due to the presence of oxides that can promote the formation of sulfate radicals and accelerate electron transfer. In particular, the CaCu_3_Ti_4_O_12_ material has shown a special interest in the field of degradation of organic pollutants by combining different AOP techniques, due to its unique and high electrical properties.

This review is composed of three main sections. The first section describes water pollution. The second section presents AOPs for water treatment. The third section describes perovskite materials, their synthesis and modification techniques, and their potential applications in water treatment.

## 2. Water Pollution

Water pollution is caused by the introduction of harmful substances or contaminants (chemicals and bacteria) into water bodies. The sources of water pollution can be natural or human-made, including industrial activities, agriculture, urbanization, and sewage disposal [13,14,15]. Water pollution can have detrimental effects on aquatic ecosystems and wildlife, as well as on human health, particularly in developing countries where access to clean water is limited. Chemicals, nutrients, pathogens, heavy metals, and plastics are some of the most common water pollutants. Addressing water pollution requires effective wastewater management and treatment, regulation of industrial activities, and sustainable agricultural practices [8,9,10,11,12,13,14,15,16,17,18,19,20]. Water pollution can have harmful effects on human health [16,17] and significantly affects environmental systems [8,18,19,20,21]. Furthermore, water sources can be contaminated by chemical, nutrient, biological, physical, and radiological pollution. Recent studies have shown that worldwide, chemical pollution is the most prevalent type of water pollution, particularly pharmaceutical pollutants.

### Pharmaceutical Pollution, Source, Occurrence

Pharmaceuticals are commonly used to prevent and treat diseases in humans and animals. They are considered emerging contaminants due to their persistent nature and potential to harm aquatic ecosystems [22,23]. There are different classes of pharmaceuticals in function of their therapeutic purpose. Antibiotics (e.g., tetracycline, ciprofloxacin, chloramphenicol), antivirals (oseltamivir and zanamivir), antidiabetics (sulfonylurea), antidepressants (alprazolam and benzodiazepines), antiepileptics (felbamate and carbamazepine), analgesics (acetaminophen, ibuprofen, and naproxen), and hormones (estriol) [24] are among the most used drugs. Many emerging pharmaceuticals have been detected in aquatic environments at varying concentrations, from ng/L to mg/L (Table 1). Despite these low concentrations, they may have very harmful effects. Pharmaceuticals may end up in wastewater treatment plants through different routes, including agricultural activities, domestic sewage, landfill sites, industrial effluents, septic tanks, urban wastewater, and even routine activities [25,26].

**Figure 1:**
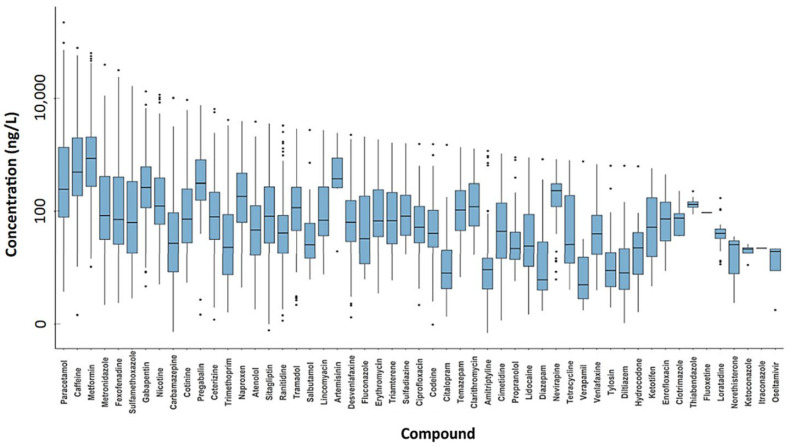
API concentrations (ng/L) in surface water samples from different countries [33].

In 2021, a global-scale study on the active pharmaceutical ingredient (API) pollution of the world’s rivers was published (Antarctica, 24 African, 24 Asian, 37 European, 6 North American, 3 Oceanian, and 9 South American rivers [33]). This study included data on 61 APIs and other compounds used in medicine and as lifestyle goods. The highest cumulative API concentrations were detected in sub-Saharan Africa, South Asia, and South America. Moreover, the most contaminated water samples in Europe originated from Madrid, Spain (mean API concentration of 17.1 μg/L and maximum concentration of 59.5 μg/L). Four compounds were detected in all continents: caffeine and nicotine (stimulant and lifestyle compounds), paracetamol (analgesic), and cotinine (nicotine metabolite). Furthermore, among the APIs measured, paracetamol presented the highest concentration in surface water (Figure 1).

**Figure 2:**
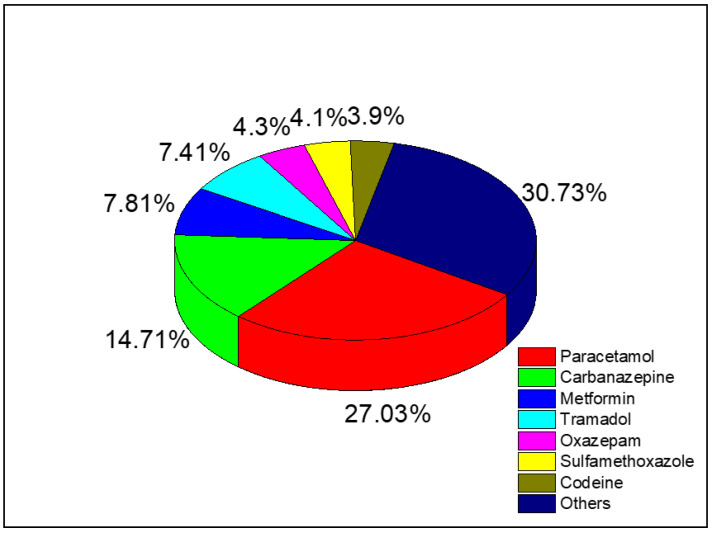
Detection rate of the indicated pharmaceuticals in France (adapted from [34]).

These results are in agreement with a previous study at different groundwater locations in France published in 2011 [34]. Specifically, paracetamol (over-the counter painkiller) was the most commonly detected emerging pollutant (27% of all analyzed samples) (Figure 2).

Due to its low cost and availability without prescription, low risk of side effects and wide use as self-medication, paracetamol is the most used drug worldwide for minor pain, headache and fever [35,36,37]. Paracetamol concentrations from 0.1 to 400 µg/L have been detected in effluent samples collected in different countries, such as 11.3 µg/L in French wastewater treatment plants [38], 150 µg/L in the USA wastewater treatment plants [39], and 11.7 µg/L in the United Kingdom wastewater treatment plants [40]. Paracetamol (Table 2) remains in the environment and can cause several effects.

These findings stress the need of effective water treatment technologies to reduce the presence of pharmaceuticals in wastewater and mitigate their potential effects on the environment and human health.

## 3. Water Treatment

Water pollution, specifically from emerging pharmaceutical contaminants, is a worldwide problem. However, the methods commonly used in wastewater treatment plants such as filtration, coagulation and sedimentation may not completely remove such pollutants, leading to their accumulation in surface water and groundwater [41,42]. Therefore, this review focuses on AOPs that can mineralize pharmaceutical products without generating harmful by-products.

AOPs are based on the generation of reactive species using various methods. These include electrochemical AOPs (anodic oxidation, electro-Fenton and photo-electro-Fenton process) based on the application of electrical energy, physical AOPs that rely on sound waves (ultrasound and microwave), UV-based AOPs (UV/O_3_ and UV/H_2_O_2_) based on UV sources for activation, catalytic AOPs (homogeneous photo-Fenton and heterogeneous photocatalysis processes) that require a catalyst, and ozone-based AOPs. These AOPs have been assessed in many studies to remove paracetamol from wastewater (list in Table 3).

### 3.1. Anodic Oxidation

Electro-oxidation is one of the simplest AOPs used for degrading organic compounds present in wastewater. Electro-oxidation requires a power supply, a cathode, an anode and an electrolyte. The anode is used to directly transfer electrons or indirectly oxidize organic compounds, generating reactive oxygen species at the anode surface for wastewater decontamination [9,10,51,52,53,54].

#### 3.1.1. Direct Oxidation

**Figure 3:**
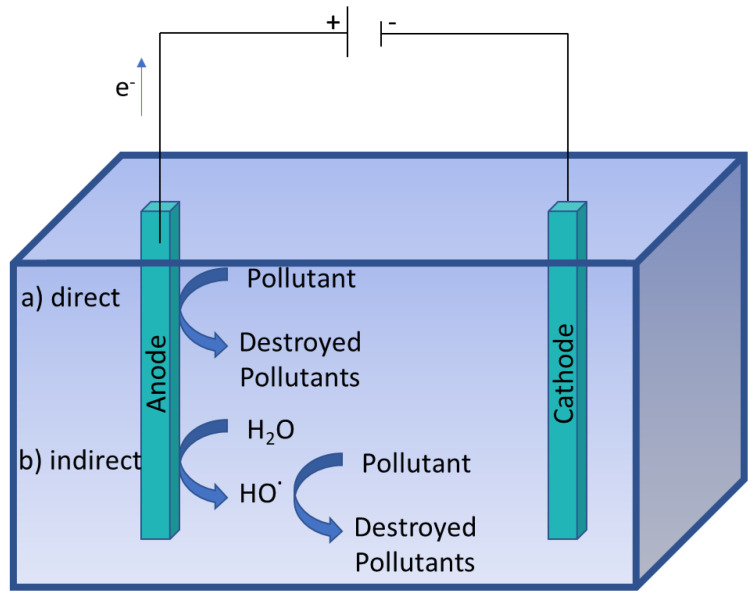
Electro-oxidation by (**a**) direct and (**b**) indirect oxidation.

In this AOP, pollutant mineralization occurs at the electrode surface or by direct electron transfer to the anode in two steps: (i) pollutant diffusion from the solution to the anode surface; and (ii) pollutant oxidation (Figure 3).

#### 3.1.2. Indirect Oxidation

In this process, a strong oxidizing agent, produced at the anode surface via an electrochemical process, diffuses into the bulk solution to oxidize and destroy the pollutant (Figure 3).

#### 3.1.3. Parameters That Influence Electro-Oxidation

**Figure 4:**
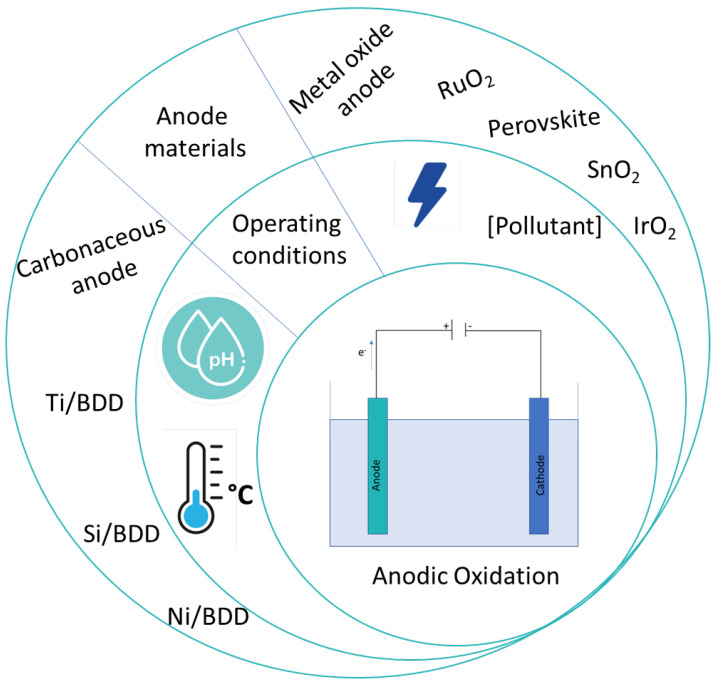
Different parameters that influence anodic oxidation.

Electro-oxidation is influenced by the anode composition and operation conditions, such as pH, temperature and currents density (Figure 4).

**Figure 5:**
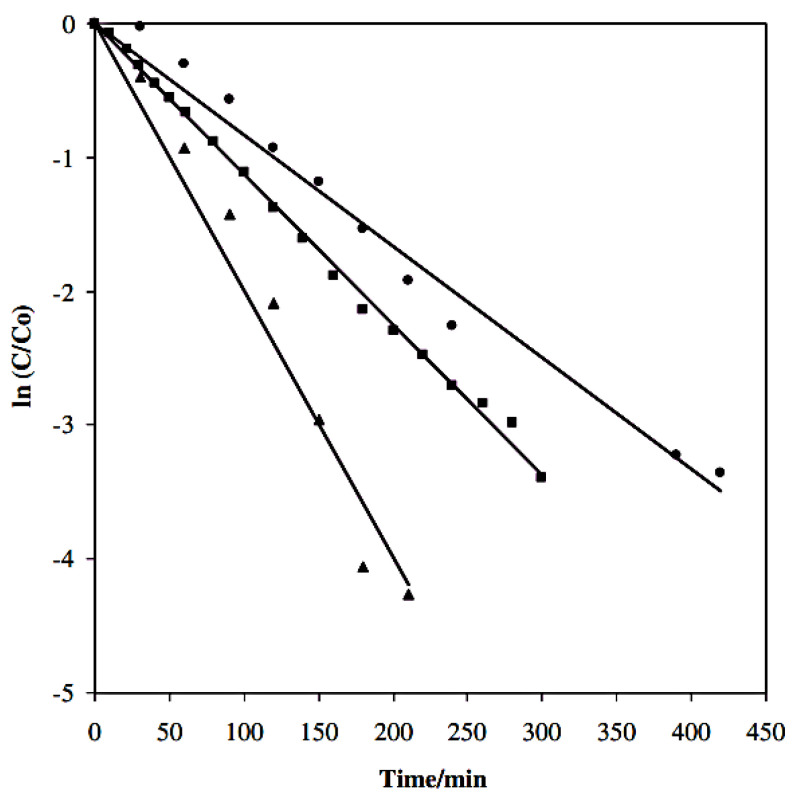
Paracetamol removal in the presence of anodes made of BDD (triangles), Ti/IrO_2_ (squares), and Ti/SnO_2_ (circles) [55], [PCM] = 1 mM and current density = 500 mA.

The choice of anodic material is critical for electrochemical reactions and significantly influences the electrocatalytic degradation efficiency. A good anode must display high electrical conductivity, catalytic selectivity and activity, high physical and chemical stability, and low cost. Waterston et al. analyzed the effect of different anodes (boron-doped diamond (BDD), Ti/IrO_2_, and Ti/SnO_2_) on the degradation of 1 mM paracetamol at an applied current of 500 mA [55]. They found that all three anodes degraded paracetamol with pseudo-first-order kinetics (Figure 5). BDD showed the highest kinetic constant: k(BDD) = 0.0218 ± 0.0013 min^−1^. Several electrode types, including graphite, SnO_2_, RuO_2_ and TiO_2_, perovskite and BDD, have been investigated and all showed significant oxygen transient progression and ability to produce enough HO^●^ radicals [56,57,58].

**Figure 6:**
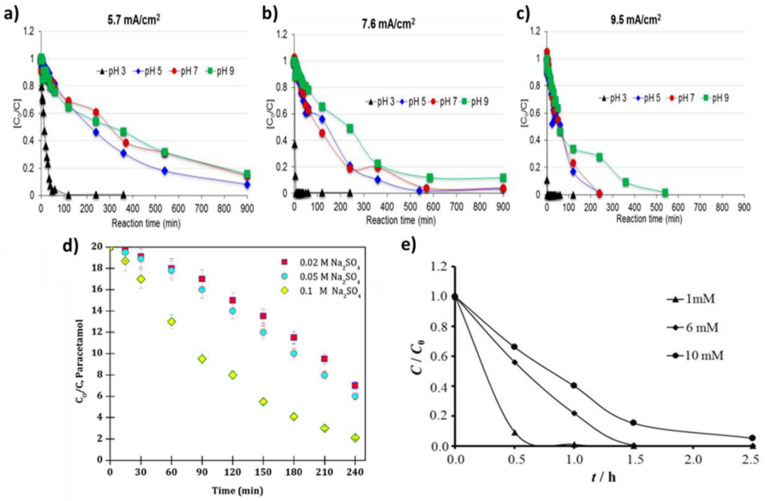
Paracetamol degradation is influenced by (**a**–**c**) pH at different current densities (**a**) 5.7 mA/cm^2^ (**b**) 7.6 mA/cm^2^ and (**c**) 9.5 mA/cm^2^ using stainless steel anode [59]; (**d**) Na_2_SO_4_ concentration using graphite anode (pH: 4.0, [PCM]: 20 mg L^−1^, current density: 5.1 mA/cm^2^) [60]; and (**e**) the initial paracetamol concentration using BDD anode (pH: 0.6, current density: 70 mA cm^−2^) [43].

Zavala et al. investigated the influence of pH and current density on paracetamol removal in the presence of an anode made of stainless steel [59]. Paracetamol was successfully degraded under all tested conditions, but the degradation process was faster at higher current densities and lower pH levels. This acceleration can be attributed to the presence of active chlorine, generated from the anodes, which oxidized the paracetamol (Figure 6a–c). Paracetamol was completely degraded in 2.5 min at pH = 3 and a current density of 9.5 mA/cm^2^. Paracetamol degradation is also influenced by the electrolyte concentration. Indeed, Periasamy et al. used three different initial concentrations of Na_2_SO_4_ (0.02, 0.05 and 0.1 mM) in an electrocatalytic system with a graphite anode as a working electrode [60]. They found that in the presence of 0.1 M Na_2_SO_4_, 90% of paracetamol was removed in 240 min (current density of 5.1 mA/cm^2^) due to the formation of sulfate radicals and stable oxidants (Figure 6d).

The degradation efficiency is also influenced by the initial pollutant concentration. Kouadio et al. studied the effect of increasing paracetamol concentrations (1, 6 and 10 mM) on the electro-oxidation process efficiency using BDD as an anode [43]. At a current density of 70 mA cm^−2^ and pH 0.6, paracetamol degradation over time was inversely correlated with its initial concentration in the solution (Figure 6e). After 1 h of electrolysis, the oxidation efficiencies were 60%, 78%, and 99% for paracetamol concentrations of 10 mM, 6 mM, and 1 mM, respectively.

#### 3.1.4. Advantages and Disadvantages of Electro-Oxidation

Electro-oxidation has been used in different studies due to its numerous advantages: (i) easy operation with simple equipment; (ii) environmentally friendly process with clean reagents (electrons) and without additional chemicals; (iii) versatile technology that can be used for the removal of a wide range of contaminants, including organic pollutants, heavy metals, and microorganisms; and (iv) need of lower pressure and lower temperatures compared with non-electrochemical alternatives [61,62,63]. The main disadvantages are: (i) the need of high energy; (ii) the use of expensive materials as electrodes that can increase the initial capital cost; and (iii) electrode fouling when materials accumulate on the electrode surface [64].

### 3.2. Photocatalysis

**Figure 7:**
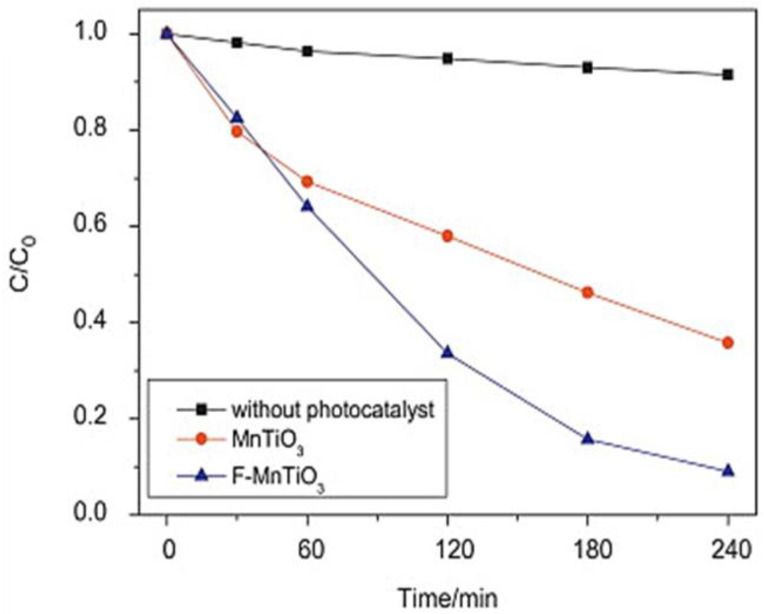
Photodecomposition efficiency of rhodamine B without photocatalyst, photocatalyst/MnTiO_3_, and photocatalyst/F-MnTiO_3_ [68].

Photocatalysis is recognized as an environmentally friendly process referred to as “green technology”. It operates by utilizing the energy from light to activate a semiconductor material. When the photocatalyst is exposed to light, it absorbs photons and generates electron–hole pairs. The excited electrons and holes can participate in various redox reactions with surrounding molecules, including the degradation of pollutants [65,66]. While TiO_2_-based materials have been extensively studied for their photocatalytic applications, there has been a growing interest in exploring ternary and other complex oxide systems as alternative photocatalysts. However, TiO_2_ has limitations, such as low sunlight absorption and limited recovery and fast recombination of photogenerated charges. The use of TiO_2_ perovskites, which can be modified by doping, is a promising method for reducing these limitations [67]. Dong et al. demonstrate the ability of F-doped MnTiO_3_ perovskite materials to degrade rhodamine B under visible light [68]. MnTiO_3_ doping improved charge separation, and increased the photocatalytic degradation by 40% compared with MnTiO_3_, as shown in Figure 7.

### 3.3. Sulfate-Radical-Based AOPs

Sulfate-radical-based AOPs have also been widely studied. In this catalytic AOP, sulfate radicals (SO_4_^●−^) are produced to degrade organic and inorganic contaminants in wastewater [69,70,71]. These radicals (Table 4) are highly reactive and can oxidize a wide range of pollutants, including pesticides, pharmaceuticals, and endocrine-disrupting compounds [72,73].

#### 3.3.1. Sulfate Activation

Methods for peroxymonosulfate/peroxydisulfate (PMS/PDS) activation are described in the following sections and summarized in Table 4.

##### Heat Activation

Heat (>60 °C) is used to activate PMS and PDS by breaking the peroxide bond to generate sulfate or mono-sulfate radicals as follows [74,75,76]:

S_2_ O_8_^2−^ → 2 SO_4_^●−^ (1)

HSO_5_ ^−^ → 2 SO_4_^●−^+ HO^●^ (2)

**Figure 8:**
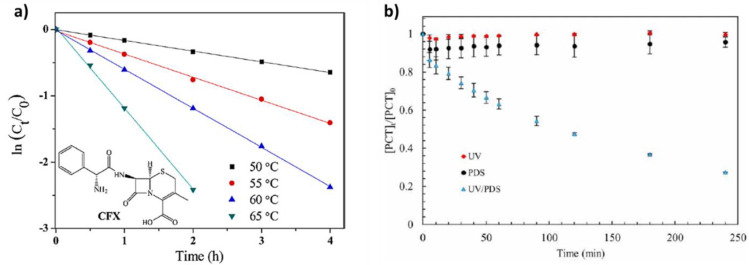
(**a**) Effect of different temperatures on cefalexin degradation ([PS]: 1.1 mM, pH: 7, and [CFX]: 100 μM) [81]; and (**b**) different systems for paracetamol degradation: UV or/and PDS (pH: 7, [PDS] = 20 mM, and [PCT] = 0.132 mM) [48].

Thermal activation of PMS/PDS has been used to degrade different emergent contaminants, such as fluoroquinolones [77], tetracycline [78], acid orange [79] and carbamazepine [80]. Qian et al. investigated whether temperature influences PS activation for cefalexin removal [81]. They found that the degradation rate increased from 47.6 to 100% by increasing the temperature from 50 to 65 °C (Figure 8a).

##### Radiation Activation

Different radiation types can be used to activate PDS and PMS, such as ultraviolet (UV), gamma rays, and ultrasound [82,83].

UV irradiation is a cost-effective activation system. It involves a photochemical process that results in the cleavage of the peroxide bonds, producing sulfate and hydroxyl radicals [71].

PMS + hν → SO_4_^●−^ + HO^●^ (3)

Similarly, the general reaction for PDS activation by UV radiation is:

PDS + hν → 2SO_4_^●−^ (4)

Dibene et al. evaluated the importance of coupling UV and PDS for paracetamol degradation [48]. They observed the highest efficiency (72.6% of paracetamol degradation) by combining UV and PDS compared with UV or PDS alone (no degradation) (Figure 8b). This confirmed that UV radiation is needed for PDS activation to increase paracetamol degradation.

Ultrasonic radiation also can activate PDS/PMS through peroxide bond cleavage, as described for UV irradiation (Equations (3) and (4) but by changing hν with ultrasound).

##### Transition Metal Ions and Metal Oxides

PMS/PDS can also be activated using various metal ions (Fe^2+^, Co^2+^, Mn^2+^, Cu^2+^, and Ni^2+^) for drug removal from water (Equations (5) and (6)).

S_2_O_8_^2−^ +M^n^ → M^n+1^ SO_4_^●−^ + SO_4_^2−^ (5)

HSO_5_^_^ +M^n^ → M^n+1^ SO_4_^●−^ + OH^−^ (6)

**Figure 9:**
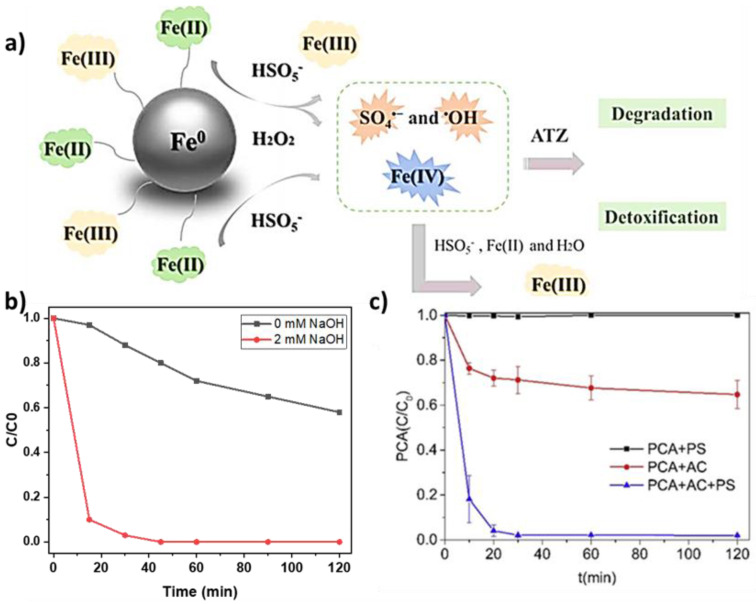
(**a**) Activation of the Fe^0^/PMS system [85]; (**b**) effect of alkaline pH on methylene blue degradation ((PMS): 60 mM and (methylene blue): 0.03 mM) (adapted from [89]); and (**c**) effect of the indicated systems on p-chloroaniline (PCA) degradation; AC, activated carbon ((PS) = 2.5 mM, (PCA) = 0.5 mM, and AC = 5 g/L) [90].

The best performance (PMS activation) was obtained with Co^2+^ [71]. These methods are categorized as homogeneous and heterogeneous in function of the catalyst form. Homogeneous systems are very efficient for the degradation of various pollutants via PMS/PDS activation. However, they can harm the ecosystems and are difficult to recycle. This limits their practical applicability. Therefore, heterogeneous catalysts have been investigated recently [84]. Li et al. studied PMS activation by Fe^0^ for atrazine removal [85]. They showed that Fe^0^ corrosion leads to the generation of Fe^2+^ ions that activate PMS to produce sulfate and hydroxyl radicals and the formation of Fe(IV) to mineralize atrazine (Figure 9a).

Tan et al. assessed PMS activation by CoFe_2_O_4_ and MnFe_2_O_4_ for paracetamol degradation (10 mg/L). Both catalysts efficiently degraded paracetamol via PMS activation by Mn^2+^-Mn^3+^/Fe^3+^-Fe^2+^ recycle and Co^2+^-Co^3+^/Fe^3+^-Fe^2+^ [86].

##### Alkaline Activation

PDS and PMS activation are influenced by the solution pH. At alkaline pH, PDS forms sulfate radicals that are then transformed into hydroxyl radicals, as described by Equations (7)–(9) [87].

S_2_O_8_^2−^ + H_2_O → 2SO_4_^●−^ + HO_2_^−^ + H^+^ (7)

S_2_O_8_^2−^ + HO_2_^−^ → SO_4_^●−^ + SO_4_^2−^ + O_2_^●−^ + H^+^ (8)

HSO_5_^−^ + OH^−^ → SO_5_^2−^ + H_2_O (9)

Qi et al. first reported the pathway of peroxymonosulfate activation by alkaline conditions [88]. The results showed the degradation of various organic substances, such as phenol, acid orange 7, and bisphenol A, using an alkaline pH for PMS activation. In addition, Yang et al. showed that PMS activation in alkaline conditions (addition of 2 mM NaOH) enhances methylene blue degradation [89] (Figure 9b). Upon PMS activation in the alkaline solution, methylene blue was completely removed in 45 min.

##### Carbonaceous-Based Materials

Carbon-based materials also can activate PDS/PMS and display several advantages, particularly a large specific surface area and cost-effectiveness. PDS/PMS activation is facilitated by the donation of electrons from carbon-based materials following Equations (10) and (11).

S_2_O_8_^2−^ + e^−^ → SO_4_^●−^ + SO_4_^2−^ (10)

HSO_5_^−^ + e^−^ → SO_4_^●−^ + OH^−^ (11)

Yao et al. showed that PDS alone cannot degrade p-chloroaniline [90], and activated carbon can degrade only 40% (Figure 9c). However, when activated carbon and PDS were combined, p-chloroaniline was fully degraded in 30 min.

##### Electro-Oxidation Activation

To decrease the amount of energy needed for electro-oxidation, the synergistic effects of hydroxyl and sulfate radicals can be combined in electro-oxidation to optimize PMS/PDS activation [91]. As activation depends on the electrode materials, Equations (12) and (13) illustrate the mechanism for cathode activation:

S_2_O_8_^2−^ + e^−^ → SO_4_^●−^ + SO_4_^2−^ (12)

HSO_5_^−^ + e^−^ → SO_4_^●−^ + OH^−^ (13)

For anode activation, a non-radical route of PMS/PDS activation leads to the formation of a special transition state (HSO_5_^−^/S_2_O_8_^2−^) with high activity for the degradation of pharmaceutical compounds [92,93].

##### Hybrid Activation

The term ‘hybrid activation’ describes the combined use of different activation methods to generate sulfate radicals. Hybrid activation improves the degradation efficiency and selectivity for target contaminants through the synergistic effect and increased reactive species formation. Recent studies showed the importance of hybrid activation for the removal of trace contaminants (Table 5).

**Figure 10:**
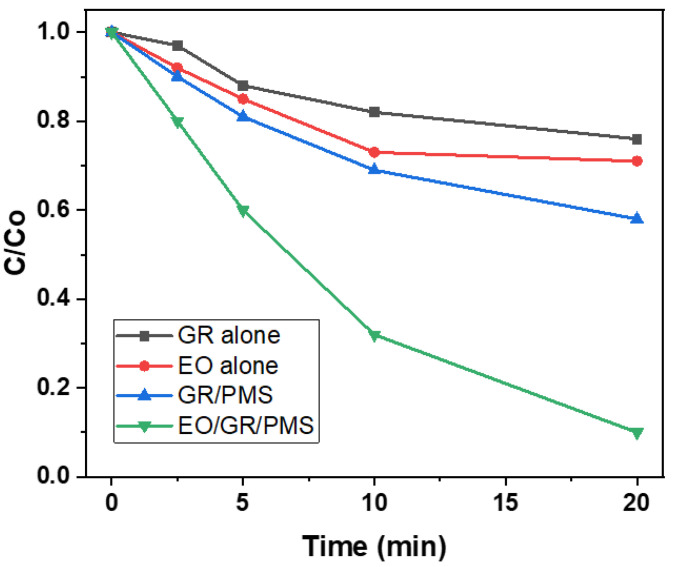
Different systems for atrazine degradation (adapted from [97]); GR, graphite; EO, electro-oxidation ((PMS) = 5 mM, current density = 100 A m^−2^, and (ATZ) = 5 μM).

In addition, Song et al. studied the combination of electro-oxidation and carbonaceous activation using graphite as an anode for PMS activation [97]. They found that after 20 min, only 24% and 29% of atrazine were degraded using graphite and electro-oxidation on their own, respectively (Figure 10). Atrazine degradation increased to 42% upon PMS activation by graphite and to 90% upon coupling electro-oxidation and graphite for PMS activation.

Various advanced methods have been employed for water treatment, with a specific focus on electro-oxidation (EO) and the sulfate-radical-based advanced oxidation process (AOP). EO utilizes a power supply, cathode, anode, and electrolyte to achieve water treatment. The performance of these methods is influenced by various parameters, including operating conditions and anode materials. In addition, the activation of sulfate radicals has been explored in this review. Among different activation methods, hybrid activation has demonstrated the highest removal efficiency for degrading trace contaminants. Notably, anode materials play a crucial role in activating the EO-PMS system. Extensive studies have emphasized the impact of transition metals present in the anode on the effectiveness of the hybrid system.

This section provides an overview of the perovskite structure, which serves as a promising candidate for anode materials. Specifically, the compound CaCu_3_Ti_4_O_12_ is highlighted due to its unique combination of two oxide materials and the numerous advantages it offers. Overall, through the exploration of electro-oxidation and sulfate-radical-based AOPs, along with the significance of anode materials and the potential of perovskite structures like CaCu_3_Ti_4_O_12_, this review sheds light on advanced techniques for efficient water treatment.

## 4. Perovskite

**Figure 11:**
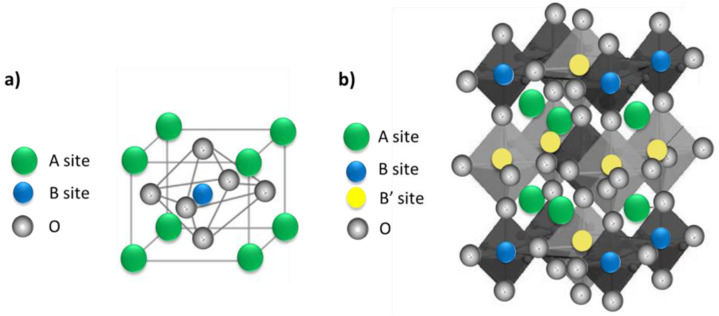
Structures of (**a**) simple perovskite, and (**b**) double perovskite.

Perovskite materials can be classified in two categories: single and double perovskite structures. The formula of single perovskite (Figure 11a) is ABX_3_ (CaTiO_3_): A and B are cations and X are anions (typically oxygen). The radius of the A ions is larger than that of B metal ions [98,99,100]. Single perovskite is used in many different applications, such as gas permeation membranes, metal–air batteries, magnetic devices, and heterogeneous catalysis [101,102]. However, single perovskite may not provide sufficient stability or performance. Therefore, recent work focused more on double perovskite structures as a promising alternative due to the improved physicochemical properties, electronic structure, stability, and efficiency [103]. The formula of double perovskites is A_2_BB’O_6_ or AA’B_2_O_6_ in which two different cations occupy the B site in the cubic lattice (Figure 11b). A cations are larger than B and B’ cations, and O is an oxygen anion. Double perovskites have a structure in which two single perovskites are present: ABO_3_ and AB’O_3_. Double perovskites can be used for various applications, such as magnetism and spintronics, electrocatalysis, catalysis, magnetocaloric, and thermoelectric [104]. In double perovskites, by changing the elemental combinations and coupling between different ions, various properties can be modified due to the different electronic configurations and ionic radius of B’ and B’’ ions [105]. For instance, complex perovskite oxides are a very versatile group of materials. CaCu_3_Ti_4_O_12_ (CCTO) is a complex double-perovskite crystal structure with a cubic group space Im-3. In CCTO, Ca^2+^ and Cu^2+^ ions are on the A and A’ sites, respectively, and Ti^4+^ ions occupy the B site [106,107]. CCTO is synthesized by incorporating Ti^4+^ and open-shell Cu^2+^ ions into the structure. A Cu^2+^ presence causes a Jahn–Teller distortion that leads to a square planar structure in the TiO_6_ octahedra [108,109,110,111,112]. Due to its many advantages, such as high dielectric constant, high chemical stability and low cost, CCTO is a versatile material for many applications.

### 4.1. CCTO Synthesis

CCTO can be synthesized using the solid state, wet chemical, combustion and microwave-assisted methods.

#### 4.1.1. Solid-State Method

**Figure 12:**
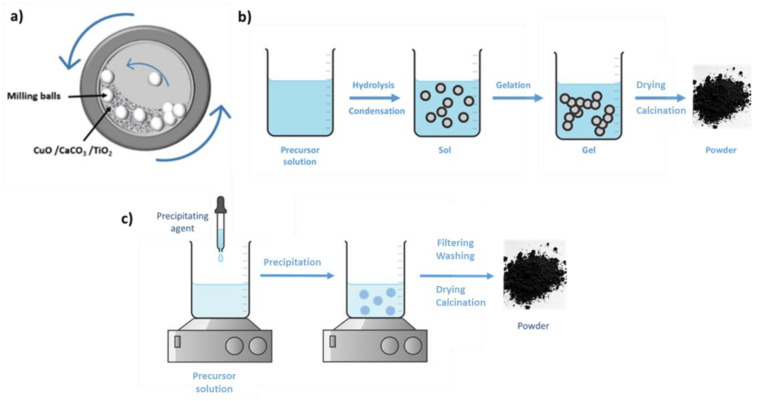
(**a**) Ball milling, (**b**) sol–gel, and (**c**) co-precipitation methods for CCTO preparation.

This approach is frequently used to fabricate CCTO ceramics. Briefly, oxides of different cations (CaCO_3_, CuO, and TiO_2_) are mixed in the appropriate stoichiometric ratio with or without a suitable liquid (acetone or ethanol) using ball-milling to obtain a fine powder. Ball milling relies on the application of mechanical force to the precursor material with grinding balls that are agitated by rotating the milling jar at a constant rate (Figure 12a) [113,114,115]. The obtained CCTO powder is heated to 900 °C (calcination) to remove volatile impurities. Equation (14) describes the reaction:

3CuO + 4TiO_2_ + CaCO_3_ → CaCu_3_Ti_4_O_12_ + CO_2_ (14)

This mechanochemical synthesis method is a fast, simple, environmentally friendly and straightforward technique for the direct synthesis of single-phase oxides at low temperatures. In this method, the starting materials can easily react with each other via a simple diffusion mechanism at room temperature. It also provides CCTO powders with a small grain size.

The mechanical reaction can be influenced by different factors, thus also affecting the properties of the obtained CCTO. These factors include the grinding time and speed and the ball/powder ratio [116,117,118,119]. Almedia et al. studied the effect of grinding time (1, 5, 10, 15, 20, 30, 60 and 100 h) on the crystalline structure of CCTO obtained, starting from CaCO_3_-3CuO-4TiO_2_ and using stainless steel vials and balls [120]. After 15 h, the peaks associated with the precursor were significantly reduced, and there was evidence of CCTO phase formation. Even after 100 h, the CCTO phase was still present, demonstrating excellent stability. Kawrani et al. used a ball mill with alumina balls and a jar to mix stoichiometric amounts of CaCO_3_, TiO_2_, and CuO (rotation speed of 350 rpm for 5 h) to prepare CCTO. After calcination at 900 °C in air for 3 h, they obtained a pure and homogeneous CCTO powder without impurities [121,122].

#### 4.1.2. Wet Chemical Methods

As an alternative to the solid-state reaction method, chemical techniques, such as sol–gel, hydrothermal, and co-precipitation, allow producing CCTO powders with enhanced purity, particle size distribution, and reactivity. Consequently, these powders require much lower sintering temperatures.

##### Sol–Gel Method

For the sol–gel method, metal alkoxides, such as calcium nitrate, copper(II) nitrate, and titanium(IV) isopropoxide, are used as precursor materials. They are hydrolyzed and condensed in the presence of a suitable solvent (e.g., ethanol) in controlled conditions (pH, temperature, and time). The resulting gel is dried, calcined, and sintered to produce the final CCTO product (Figure 12b) [123,124,125].

Liu et al. used the sol–gel method to prepare fine CCTO powders. First, they dissolved tetrabutyl titanate, calcium nitrate, and copper(II) nitrate (in stoichiometric amounts) in ethanol [126]. Second, they aged the gel at room temperature for 2 h followed by drying at 120 °C. Third, they obtained a black CCTO powder by calcination of the gel precursor in air at 900 °C. Pang et al. investigated different calcination temperatures (600, 700, 800 and 900 °C) [127]. To prepare CCTO nanoparticles with the sol–gel method, they dissolved stoichiometric amounts of calcium acetate hydrate and copper acetate hydrate in acetic acid. Then, they added this solution, drop by drop, into a solution that contained a titanium precursor. After the addition of ethylene glycol and forming amide to stabilize the solution, they stirred it for 5 min. They found that CCTO calcined at 900 °C exhibited the greatest photodegradation efficiency.

##### Co-Precipitation

The co-precipitation method, described in Figure 12c, requires the simultaneous precipitation of cations in the form of hydroxides or carbonates from their respective soluble salts, followed by calcination to form the desired oxide. Briefly, the precursor solution is prepared by mixing copper nitrate, calcium nitrate, and titanium tetraisopropoxide (stoichiometric amounts) in a solvent. Then, a precipitating agent, such as ammonium hydroxide or sodium carbonate, is added to the solution to initiate the precipitation of the cations in the form of hydroxides or carbonates. This is followed by filtration, washing, drying and calcination [128,129].

Barbier et al. prepared CCTO using this method. First, they dissolved metal chlorides (CaCl_2_, TiCl_3_, and CuCl_2_·2H_2_O) in water and then added them to the precipitating agent (oxalic acid mixed in ethanol) [130]. This was followed by calcination in air at 950 °C for 10 h to obtain the CCTO powder.

Kumari et al. produced CCTO using the co-precipitation method, as explained by Equation (15) [131]:

CaCl_2_ + 3Cu(NO_3_)2.3H_2_O + 4TiCl_4_ + 19NaOH → CaCu_3_Ti_4_O_12_ + 19NaCl + 6HNO_3_ + 16 H_2_O (15)

Briefly, they dissolved calcium chloride (CaCl_2_) and copper nitrate trihydrate (Cu(NO_3_)_2_·3H_2_O) in water, and then added a titanium tetrachloride (4TiCl_4_)-HCl saturated solution. After stirring, they added the obtained solution, drop by drop, to an aqueous solution of 0.8 M NaOH that was stirred at a constant speed. This resulted in a milky blue color solution. Then, they washed the obtained precipitation, followed by pulverization into a fine powder and calcination to obtain the CCTO powder.

#### 4.1.3. Combustion System

The combustion synthesis method (or self-propagating high-temperature synthesis method) is a cheap approach to fabricate homogeneous nanopowders and single-phase materials, such as ceramics, catalysts, composites, alloys, intermetallics, and nanomaterials. The method is based on a self-sustaining solid flame-combustion reaction for a short period, and does not need the high reaction temperatures that are typically required for ceramic synthesis. Instead of placing the sample in a furnace, high temperature is achieved via an exothermic chemical reaction between a fuel and an oxidant present in the precursor solution that maintains the high temperature for the time required to form the final product (i.e., 30–45 s). During this chemical reaction to produce heat, a large amount of gas is released, leading to the synthesis of a nanosized, porous, and foamy product [109,132].

Kumari et al. used metal nitrates, titanium isopropoxide, anhydrous citric acid, and acetyl acetone as raw materials. The auto-combustion involved mixing citric acid dissolved in de-ionized water with the titanium precursor dissolved in acetyl acetone. This was followed by an addition to the titanium solution of copper nitrate trihydrate and calcium nitrate tetrahydrate dissolved in de-ionized water. The solution was stirred and after the addition of ammonia, it was heated to allow for water evaporation and the formation of a viscous blue gel. Then, a porous structure was obtained by heating the gel at 450 °C for 15 min, followed by grinding into a fine powder and heating at 950 °C for 8 h to obtain oxides. The obtained CCTO powder contained some impurities, such as CaTiO_3_ and CuO, that can affect its properties. Additionally, the prolonged heat treatment caused the formation of CuO that led to an increase in the grain size to 600 nm [131].

#### 4.1.4. Microwave-Assisted Synthesis

Microwave energy may be used at the place of calcination to prepare pure-phase CCTO powders. The microwave heating process generates heat rapidly and uniformly throughout the reaction vessel, resulting in a shorter reaction time and lower processing temperature. Microwave-assisted synthesis relies on the heating effect generated by the microwave energy. This effect is primarily due to the interaction of microwaves with the polar molecules present in the precursor solution [133,134].

Yu et al. used the microwave-assisted solid-state method to fabricate CCTO pellets. Briefly, they mixed the precursors (CaCO_3_, CuO and TiO_2_) in a ball mill for 24 h and pressed them into pellets [135] that were calcinated by microwave heating for 30 min. The obtained ceramics displayed higher dielectric constant values than ceramics prepared using conventional synthesis methods, even when sintered in the same conditions.

CCTO synthesis methods (sol–gel, microwave, solid state, combustion, and co-precipitation) play a crucial role in determining its properties. Sol–gel methods produce large amounts of CCTO with controlled composition, but require long reaction times, expensive starting materials and the production of secondary phases due to the used solvent [136,137]. Combustion methods allow for better controlling the precursor stoichiometry, but not the morphology. Moreover, with these techniques, contaminants are formed due to the presence of carbonaceous residues [138]. The solid-state method has many advantages (simplicity, cost-effectiveness, scalability, and high-temperature stability), but does not allow for controlling the composition and morphology well [139].

### 4.2. Perovskite Modifications

In addition to the synthesis parameters, researchers have also explored various methods to modify CCTO properties, particularly doping with different metal ions and changing the atmosphere treatment. Metal doping can alter CCTO electronic and structural properties and improve its performance in various applications. Changing the atmosphere treatment, such as oxygen, nitrogen, and hydrogen, also significantly affects CCTO properties. These modifications have shown promising results for improving CCTO performance in various applications, making it a promising material for future research and development.

#### 4.2.1. CCTO Doping

Doping is a commonly used method to modify CCTO properties (i.e., dielectric constant, dielectric loss, electrical conductivity). Various types of dopants, such as Fe, Co, and Mn, have been tested. Indeed, the dopant type and concentration can significantly influence CCTO properties, and the optimal doping conditions may vary in function of the intended application. CCTO can be doped with different elements (one or two elements) and at different sites (Ca, Cu, and Ti) to modify its features. Table 6 lists the results of some of the many studies on CCTO doping.

#### 4.2.2. Atmosphere Treatment of CCTO

CCTO can also be treated in different atmospheres, such as oxygen, nitrogen, and hydrogen, to modify its properties. However, the literature on this topic is relatively limited, and the effects of these treatments are not fully understood. Some articles reported promising results in terms of enhancing CCTO specific properties, but more research is needed to determine the optimal conditions for each application [153,154,155]. Damas et al. [156] found that in the presence of nitrogen, CCTO decomposes into Cu_2_O, TiO_2_ and CaTiO_3_. Zaho et al. [157] studied the effect of different treatments (air, oxygen, and nitrogen) on CCTO composition by X-ray diffraction. In the as-prepared CCTO sample, they observed the presence of the CCTO phase and peaks related to CuO. Upon oxygen and air treatment, they could no longer detect the CuO peaks, only the CCTO phase. On the other hand, after nitrogen treatment, they observed different peaks, including the CuO, CaTiO_3_, and TiO_2_ peaks, confirming CCTO decomposition upon nitrogen treatment.

In energy storage applications, an oxidizing atmosphere increased the CCTO storage energy density from 7.7 to 26.5 kJ/m^3^, decreased the direct current loss, and increased the grain boundary activation energy to 0.85 eV.

To summarize, CCTO proprieties can be modified by different methods. CCTO can be doped with different metals (e.g., nickel, tungsten, and cobalt) to modify its resistance, grain size, and dielectric constant. These modifications improve its performance for various applications. Changing the atmosphere treatment also significantly influences CCTO properties. The atmosphere treatment leads to CCTO decomposition into other heterostructure composites that can be used in many applications. The next section presents some applications of CCTO materials.

### 4.3. CCTO Applications

**Figure 13:**
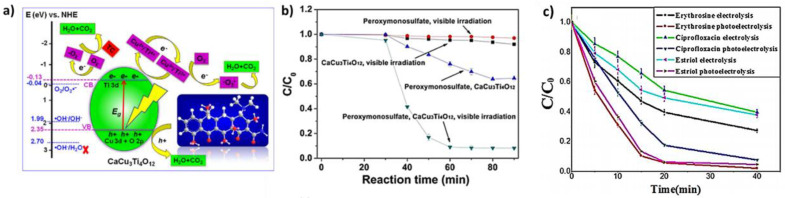
(**a**) Mechanism for the degradation of tetracycline [163]; (**b**) different systems for the degradation of ibuprofen([ibuprofen]: 20 mg L^−1^, and KHSO_5_/ibuprofen molar ratio of 5:1) [164]; and (**c**) the degradation of erythrosine, ciprofloxacin, and estriol using electrolysis and photoelectrocatalysis [165].

CCTO is a ceramic material that displays a high dielectric constant, low dielectric loss, high temperature stability, and nonlinear current–voltage characteristics [108,109,110,111,112]. Thanks to these features, CCTO is a promising material for a range of applications, such as sensors [158], hydrogen production [122], supercapacitors [159,160], microwave devices and antennas [161], water splitting applications [110] and water treatment [162]. Table 7 summarizes the CCTO applications for water treatment. Indeed, CCTO ceramics have been investigated for the photocatalytic degradation of pollutants at a lab scale. For example, Halili et al. showed that 99.1% of 100 mL of the 1.0 × 10^−5^ mol L^−1^ tetracycline solution was degraded in the presence of 40 mg CCTO [163] after 30 min of irradiation (420 nm). Figure 13a describes the mechanism proposed for tetracycline degradation.

CCTO materials can also be used in hybrid systems and for the activation of sulfate radicals. Zhu et al. studied ibuprofen degradation in the presence of CCTO powder [164]. They used different systems to determine the best combination of CCTO, PMS and visible light. After 60 min, the binary systems’ visible light/CCTO and visible light/PMS gave the lowest ibuprofen degradation rates (8.1% and 3.1%, respectively). This rate increased to 35.5% in the presence of CCTO and PMS. However, the best degradation rate (91.8%) was obtained with the PMS/CCTO/visible light system, indicating the importance of coupling different activation systems (Figure 13b). In addition, the efficacity of combining photocatalysis with electrocatalysis for the degradation shows the degradation erythrosine, ciprofloxacin, and estriol, as evaluated by Kushwaha et al. Under photoelectrocatalytic degradation, due to the generation of more OH radicals for catalysis and effective charge carrier separation by a photocurrent, the kinetic decay constants for all three pollutants were found to be higher (Figure 13c).

## 5. Future Perspectives

After confirming the efficiency of CCTO materials in the laboratory for degrading different pollutants, it will be important to investigate their performance using real water samples. Indeed, real samples may contain other impurities and contaminants that can affect the performance of CCTO materials. These impurities and contaminants present in real water can include organic matter, heavy metals, inorganic ions, and various organic pollutants. The presence of such substances can introduce challenges and complexities in the degradation process, potentially affecting the reaction kinetics and overall performance of the CCTO materials [167,168]. Hence, it is essential to conduct experiments using real water samples to simulate realistic conditions and evaluate the practical effectiveness of the CCTO materials. By studying the impact of real water on the degradation capabilities of CCTO, researchers can gain valuable insights into the material’s performance under more representative conditions, ensuring its suitability for real-world water treatment scenarios. Furthermore, investigating the quenching effect caused by organic matter in the water samples is particularly important. Organic matter can act as scavengers or quenchers, competing with the target contaminants for reactive species generated during the degradation process. Understanding how organic matter affects the degradation efficiency of CCTO materials is crucial for optimizing their performance and developing practical strategies to mitigate any quenching effects. Some studies described the effect of real water in AOPs using different materials, such as titanante [167]; however, no study has assessed the effects of real water samples on CCTO efficiency.

To further enhance the practical applicability of CCTO materials in water treatment, it is important to investigate their performance in continuous processes at a larger scale. Lab-scale experiments provide valuable insights into the material’s efficiency, but translating these results to continuous operations is essential for real-world implementations [169,170]. Scaling up the CCTO-based water treatment processes requires considering various factors such as reactor design, flow rates, residence time, and operational parameters. The design of continuous systems should ensure efficient contact between the water and CCTO material, allowing for effective pollutant degradation while maintaining a stable and consistent performance over extended periods. Continuous processes also enable the evaluation of the long-term stability and durability of the CCTO materials under realistic conditions. Factors like fouling, material degradation, and the accumulation of by-products need to be assessed to ensure the sustained performance and reliability of the system. Moreover, process optimization becomes crucial when transitioning from lab-scale to larger-scale continuous operations. This involves optimizing parameters such as CCTO material loading, flow rates, and operational conditions to maximize the treatment efficiency while minimizing energy consumption and operational costs [170,171]. Pilot-scale or full-scale demonstrations should be conducted to validate the performance of CCTO materials in continuous water treatment processes at a larger scale. These demonstrations provide insights into the scalability, economic feasibility, and reliability of the technology, allowing for a comprehensive assessment of its potential for commercial implementation.

Doping CCTO with cobalt offers several advantages for water treatment such as improving the activation of sulfate radicals. However, this approach is limited by cobalt leakage into the solution and CCTO membrane instability. To address these limitations, the membrane can be stabilized using various methods, such as a thin coating of the membrane surface by an atomic layer deposition or by a thermal treatment of the membrane after its use.

In addition, CCTO has many interesting advantages for numerous applications, specifically for water treatment and in various fields. Therefore, it is important to further study the potential of CCTO membranes in different conditions, such as electro-Fenton, or by coupling different processing techniques.

Electro-oxidation processes require a significant amount of electrical energy to drive the oxidation reactions. This could be addressed by using solar energy. CCTO contains TiO_2_ and copper. TiO_2_ is the most promising phase for photocatalytic applications, particularly when using UV light. Moreover, the presence of copper in CCTO facilitates the shift in TiO_2_ adsorption to visible light wavelengths [172]. Therefore, CCTO could be used in solar-energy-based pollutant removal processes that are more sustainable and environmentally friendly. However, more research and development are needed to optimize CCTO properties for various applications, such as enhancing its photocatalytic efficiency, electro-Fenton process, and developing novel processing techniques to improve its stability and performance. Finally, a crucial step would be transitioning to a continuous system that closely simulates industrial conditions.

## 6. Conclusions

This review described the harmful effects of water pollution on human health and the environment and the detection of various pollutant types (physical, chemical, nutrient, and radioactive) in wastewater. Among them, chemical pollution, specifically emerging pollutants, is particularly concerning due to the persistence of such molecules. Paracetamol presents the highest concentration in surface water worldwide. Similarly, in France, paracetamol was the most commonly detected (27%) emerging pollutant. AOPs could be part of the solution to this issue because they can mineralize persistent compounds. Many studies showed that different AOPs can remove paracetamol. In this review, particular attention was focused on electro-oxidation and the parameters affecting its performance. Sulfate-based AOPs and their activation methods for the formation of sulfate radicals were also discussed. The hybrid activation process (electro-oxidation/PMS) combines the synergic effect of the two systems for the degradation of water pollutants. In these systems, the anode material strongly influences the performance. This review showed that the CCTO perovskite material has several advantages, such as a high dielectric constant, low dielectric loss, high-temperature stability, and nonlinear current–voltage characteristics. The synthesis method, doping, and atmosphere treatment significantly influence the CCTO performance. Doping decreases the dielectric loss and increases the grain boundary size. Furthermore, nitrogen treatment leads to CCTO decomposition into TiO_2_, CaTiO_3_, and CuO. CCTO materials can reduce emerging compounds using different systems, such as photocatalysis, PMS activation by light, and photo-electrocatalysis. The future trend in the field of water treatment applications is to develop continuous systems for water treatment by exploring synergistic approaches (electro-oxidation/PMS) to enhance the overall efficiency and degradation capabilities. In addition, extensive studies should be performed on the performance of CCTO materials and technologies using real water samples to take into account the presence of impurities and variations in water composition. Better understanding the degradation mechanisms and the formation of harmful by-products will help to design targeted treatment strategies and optimize the performance of water treatment systems.

## Figures and Tables

**Table 1:** Pharmaceutical compounds detected in different water samples.

Water Sample	Country	Compounds Detected	Concentration (ng/L)	Reference
Drinking water	Malaysia	Amoxicillin	0.31	[27]
		Ciprofloxacin	0.32	
		Triclosan	0.36	
Surface water	Lagos, Nigeria	Acetaminophen	24	[28]
		Naproxen	18	
		Carbamazepine	9	
River	Amieira, Portugal	Acetaminophen	173.91	[29]
		Ibuprofen	72.14	
		Carbamazepine	49.37	
River	Mzundusi, South Africa	Acetaminophen	153	[30]
		Ciprofloxacin	14	
River	Lambro, Italy	Acetaminophen	18.8	[31]
		Naproxen	62.4	
Surface water	China	Acetaminophen	41.3	[32]
	USA		10.8	
	Europe		80.1	

**Table 2:** Paracetamol characteristics.

Paracetamol 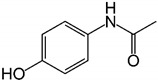	Chemical name	Acetaminophen
	Chemical formula	C_8_H_9_NO_2_
	Molecular weight	151.163 g/mol
	Solubility	14 g/L at 25 °C
	Density	1.293 g/cm³ at 25 °C
	Activity	Analgesic (pain reliever) Antipyretic (fever reducer)
	Cas number	103-90-2
	Cost	0.573 EUR/g

**Table 3:** AOPs recently tested for paracetamol (PCM) removal.

AOP Type	Process	Conditions	PCM Degradation or TOC Removal	Reference
Electrochemical AOPs	Anodic oxidation	PCM concentrations: 1 mM Electrolyte: 0.3 M H_2_SO_4_ pH = 3 Current density: 70 mA/cm^2^ Anode: BDD Cathode: zirconium plate	99% in 1 h	[43]
	Electro-Fenton	PCM concentration: 0.1 Mm Electrolyte: 50 mM Na_2_SO_4_ pH = 3 Current density: 20mA/cm^2^ Catalyst: 0.2 mM FeSO_4_·7H_2_O Anode: platinum foil Cathode: porous carbon felt	51% of TOC removal in 2 h	[44]
	Photo-electro-Fenton	PCM concentration: 5 mM pH = 3 Current density: 113 A/m^2^ UVA lamps Catalyst: 0.1 mM Fe^2+^ and 15 mM H_2_O_2_ Anode: titanium coated with RuO_2_/IrO_2_ Cathode: stainless steel	99% in 60 min	[45]
Physical AOPs	Ultrasound	PCM concentration: 25 ppm Ultrasound irradiation: 20 kHz and 40 W	12% in 180 min	[46]
UV-based AOPs	UV/H_2_O_2_	UV irradiation at 254 nm 5 mg/L H_2_O_2_	100% in 30 min	[47]
	UV/Peroxydisulfate	PCM concentration: 0.132 mM PDS concentration: 20 mM pH = 6 Lamp power 30 W	92% in 240 min	[48]
Catalytic AOPs	Fe_3_O_4_/Peroxymonosulfate	PCM concentration: 10 ppm PMS concentration: 0.2 mM Fe_3_O_4_ concentration: 0.8 g/L	75% in 120 min	[49]
Ozone-based AOPs	O_3_	PCM concentration: 5.3 × 10^−3^ mol/dm^3^ pH = 2 Ozone flow rate: 36 dm^3^/h	40% in 120 min	[50]

**Table 4:** Proprieties of sulfate radicals.

SO_4_^●−^	Oxidation capacity	2.5–3.1 V
	Half-life	30–40 μs
	pH range	2.0–8.0
	Obtained from	Peroxymonosulfate (PMS, HSO_5_^−^)
		Peroxydisulfate (PDS or PS, S_2_O_8_^2−^)
	Activated by	UV irradiation
		Heat
		Transition metal ions and metal oxides
		Carbon-based catalysts
		Hybrid activation

**Table 5:** Hybrid activation for the removal of pharmaceutical compounds.

Pollutant	Process	Pollutant Concentration	Electrode Materials	Current/Sulfate Concentration	Removal Percentage	Ref.
Cefadroxil	EC/PMS	30 mg/L	Ti/La_2_O_3_-PbO_2_ anode	PMS: 5 mM Current density: 10 mA/cm^2^	100% in 60 min	[11]
Iohexol	EC/PMS	10 mg/L	LaCoO_3_-V_O_	PMS: 0.1 mM Current density: 10 mA/cm^2^	90% in 10 min	[94]
Ciprofloxacin	Photocatalysis/PMS	7.00 mg/L	CeFeO_3_ with nitrogen-doped carbon quantum dots	PMS: 4.90 mM Catalyst: 0.80 g/L λ: 400–800 nm	92.5% after 2.5 min	[95]
Tetracycline	Photocatalysis/PMS	10 mg/L	CaTiO_3_-CaFe_2_O_4_	PMS: 1 mM λ > 420 nm	94.6% after 120 min	[96]

**Table 6:** Different examples of CCTO doping.

Doping Element	Site	Ratio	Synthesis Method	Results	Ref.
Tungsten (W)	Ti-site CaCu_3_Ti_4−x_W_x_O_12_	x = 0.01, 0.03, and 0.05	Flame synthesis	Decreased resistance of grain boundaries. Increased average grain size.	[140]
Selenium (Se)	Cu-site CaCu_3−x_Se_x_Ti_4_O_12_	x = 0, 0.1, 0.2, and 0.3 mM	Sol–gel	Increased dissipation factor. Reduced dielectric constant. Decreased AC conductivity.	[141]
Nickel (Ni)	Cu-site CaCu_3−x_Ni_x_Ti_4_O_12_	x = 0.00, 0.10, 0.20, 0.25, 0.35, and 0.40	Solid-state reaction	Ni doping in the grains increases the Cu-rich phase amount within the intergranular phase. Reduced dielectric loss. At the beginning, Ni doping occurs more at the Cu site than Ti site. As the amount of Ni increases >0.25, Ni doping is also observed at the Ti site.	[142]
	Cu-site CaCu_2.9_Ni_0.1_Ti_4_O_12_ Ti-site CaCu_3_Ti_3.9_Ni_0.1_O_12_		Sol–gel	No structural change. Increased dielectric constant and dielectric loss at the Ni-doped Cu site than at the Ni-doped Ti site	[143]
Manganese (Mn)	Cu-site CaCu_3−x_Mn_x_Ti_4_O_12_	x = 0.0, 0.01, 0.02, 0.03, 0.04, and 0.05	Solid-state reaction	x = 0.01 and 0.02 → relaxation time and activation energy slightly decreased. x ≥ 0.03 → dielectric response significantly reduced.	[144]
Strontium (Sr)	Cu- site CaCu_3−x_Sr_x_Ti_4_O_12_	x = 0, 0.05, 0.1, 0.2, and 0.4	Solid-state reaction	Dielectric loss decreases when the x value increased. Higher permittivity, suitably low dielectric loss, and improved DC bias voltage stability.	[145]
Nickel and Strontium	Ca-site and Cu-site Ca_1−x_Sr_x_Cu_3−y_Ni_y_Ti_4_O_12_	x = y = 0; x = 0.1, y = 0; x= 0, y = 0.1 ; and x = 0.1, y = 0.1	Solid-state reaction	Sr^2+^ and/or Ni^2+^ doping increases the grain size. Highest grain boundary resistance. Increased nonlinear coefficient values. Decreased breakdown electric field and leakage current. Best conductive grain and insulating grain boundary.	[146]
Gallium (Ga)	Ti-site CaCu_3_Ti_4−x_Ga_x_O_12_	x = 0, 0.01, 0.05, and 0.1	Solid-state reaction	Increased mean grain size. x = 0.25 mol% of Ga^3+^ → dielectric constant increases from 5439 to 31,331 → loss tangent decreases from 0.153 to 0.044.	[147]
Europium (Eu)	Ca-site Ca_1−x_Eu_2/3x_Cu_3_Ti_4_O_12_	x = 0, 0.1, 0.3, 0.6, and 0.9	Solid-state reaction	Lack of oxygen vacancies. Increased dielectric loss. Decreased dielectric constant.	[148]
Chromium (cr)	Ti-site CaCu_3_Ti_4−x_Cr_x_O_12−x/2_	x = 0, 0.01, 0.02, and 0.03	Solid-state reaction	Increased complex permittivity. Increased DC conductivity. Movements of oxygen vacancies at the grain boundaries.	[149]
Cobalt (Co)	Cu-site CaCu_3−x_Co_x_Ti_4_O_12_	x = 0.00, 0.05, 0.10, and 0.20	Solid-state reaction	x = 0.05: lowest dielectric loss, highest grain boundary resistance, and highest cation vacancies. Co doping at the Cu site, initial presence of Co^2+^. x ≥ 0.1 Co doping at Cu and Ti sites and presence of Co^3+^/Co^2+^.	[150]
	Ti-site CaCu_3_Ti_4−x_Co_x_O_12_	X = 0, 0.2, and 0.4	Solid-state reaction	Grain boundaries: Cu and Co phases. Low magnetic loss. Ferromagnetism in co-doped CCTO: super-exchange interactions via Cu-O-Co-O-Cu.	[151]
	Cu-site CaCu_3−x_Co_x_ Ti_4_O_12_	x = 0.00, 0.05, 0.10, and 0.20	Sol–gel modified	Decreased grain size. Decreased leakage current. X = 0.05 high dielectric constant. Dielectric loss and high nonlinear coefficient.	[152]

**Table 7:** CCTO applications in AOPs in different conditions.

	Pollutant	Process	Conditions	Results	Ref.
CCTO ceramic pellets	Erythrosine	Photocatalytic	10 mL of 10 mg/L ciprofloxacin, 10 mg/L erythrosine and 1 mg/L of estriol visible light 150 W	A total of 77% of degradation after 60 min	[165]
	Ciprofloxacin			A total of 64% of degradation after 60 min	
	Estriol			A total of 51% of degradation after 60 min	
CCTO photo-anode	Erythrosine	Photo-electrocatalysis	Cathode: platinum wire Reference electrode: SCE Bias potential: from 0.5 V to 1.5 V Visible light: 100 mW cm^−2^	A total of 100% of degradation after 40 min	[165]
40.0 mg of CCTO	Tetracycline	Photocatalytic	100 mL of 1.0 × 10^−5^ mol L^−1^ tetracycline Light intensity: 500 mW cm^−2^	A total of 99.1% of degradation after 30 min higher constant k = 1.1 × 10^−1^ min^−1^	[163]
CCTO powder	Ibuprofen	Visible light/peroxymonosulfate	0.5 mM PMS 100 mL of 20 mg L^−1^ ibuprofen 300 W xenon lamp	A total of 91.8% of degradation after 60 min	[164]
0.05 g CCTO powder	Rhodamine B	Photocatalytic	200 mL of 5 ppm Rhodamine B UV irradiation λ = 255 nm	A total of 52% of degradation after 40 min of UV irradiation	[166]

## Data Availability

Not applicable.
